# A Doctor’s Value

**Published:** 1855-11

**Authors:** 


### A DOCTOR'S VALUE,

Is not correctly estimated by the number of lives saved or lost in his individual practice, but by the results of his investigations of the great principles of human health and life. He may be a week, or a month, or a year in restoring one man to health, or conducting another, by skilful appliances, to a comparatively painless grave; yet, in much less time he may, in the retired quiet of his own office, eliminate a truth, which shall save millions from a premature grave, and give to millions of others hope, and health, and comfort and bread, "and the poor man's home once more be filled with smiles and gladness. We must have a dozen great markets, free as the air we breathe, and a hydrant at one of every four corners of the streets. No nation can be happy, and no city can be governed without the bayonet and the prison, where wholesome food and pure water are not within the reach of every industrious honest man." Such is the close of a suggestive article in the "*Scalpel*" for September. Truer words have never been written. The impositions and extortions of the hucksters, "the middle men" of New York, and the barefaced cheateries of the *corner groceries*, as a class, are well exposed in that caustic article; by the machinations of the former, good substantial bread and meat are placed beyond the reach of thousands of the laboring and deserving families of this great city, in consequence of their high price, while by the latter, refuse and rotting articles are supplied, in short measure, to the hardly toiling poor—at a hundred per cent. beyond their intrinsic, legitimate value.

We say with Dr. Dixon, "let the city markets be numerous, roomy and free," and we add, *free only to those who bring the produce from their own farms or gardens.*

And further than that, we suggest, as to articles which are purchased in bulk for winter use, such as flour, potatoes, turnips, apples, butter, lard and the like, that there be contributions of five or ten families, to send a competent person a hundred, or two or three hundred miles from the city, where good articles, of the above named, can be procured, and delivered at the door in New York, one hundred per cent. less than the city price. A fact explains fully our meaning. The "*Boston Traveller*" states that some weeks since a gentleman of Boston was travelling in the West, and while at Chicago purchased half a dozen barrels of fine flour, for his own use, at $5 87 a barrel. He sent it to Boston, and the extreme cost, delivered at his house there, was $7 75 a barrel. At that time the same brand of flour was selling there at *fourteen dollars* a barrel, or for nearly double what the gentleman's cost him.

